# Peak cortisol response to corticotropin-releasing hormone is associated with age and body size in children referred for clinical testing: a retrospective review

**DOI:** 10.1186/s13633-015-0018-y

**Published:** 2015-10-22

**Authors:** Mary Ellen Vajravelu, Jared Tobolski, Evanette Burrows, Marianne Chilutti, Rui Xiao, Vaneeta Bamba, Steven Willi, Andrew Palladino, Jon M. Burnham, Shana E. McCormack

**Affiliations:** Division of Endocrinology and Diabetes, The Children’s Hospital of Philadelphia, 3401 Civic Center Blvd, Suite 11NW, Philadelphia, USA; Center for Biomedical Informatics, The Children’s Hospital of Philadelphia, 3535 Market St, Philadelphia, PA 19104 USA; Department of Biostatistics and Epidemiology, University of Pennsylvania, 635 Blockley Hall, 423 Guardian Drive, Philadelphia, PA 19104-6021 USA; Global Innovative Pharma, Pfizer, Inc, 500 Arcola Road, Collegeville, PA 19426 USA; The Children’s Hospital of Philadelphia, Division of Rheumatology, 10103 Colket Building, 34th & Civic Center Blvd, Philadelphia, PA 19104 USA

**Keywords:** Adrenal insufficiency, Corticotropin-releasing hormone (CRH) stimulation test, Exogenous glucocorticoid exposure, Adrenal stimulation testing, Cortisol

## Abstract

**Background:**

Corticotropin-Releasing Hormone (CRH) testing is used to evaluate suspected adrenocorticotropic hormone (ACTH) deficiency, but the clinical characteristics that affect response in young children are incompletely understood. Our objective was to determine the effect of age and body size on cortisol response to CRH in children at risk for ACTH deficiency referred for clinical testing.

**Methods:**

Retrospective, observational study of 297 children, ages 30 days – 18 years, undergoing initial, clinically indicated outpatient CRH stimulation testing at a tertiary referral center. All subjects received 1mcg/kg corticorelin per institutional protocol. Serial, timed ACTH and cortisol measurements were obtained. Patient demographic and clinical factors were abstracted from the medical record. Patients without full recorded anthropometric data, pubertal assessment, ACTH measurements, or clear indication for testing were excluded (number remaining = 222). Outcomes of interest were maximum cortisol after stimulation (peak) and cortisol rise from baseline (delta). Bivariable and multivariable linear regression analyses were used to assess the effects of age and size (weight, height, body mass index (BMI), body surface area (BSA), BMI z-score, and height z-score) on cortisol response while accounting for clinical covariates including sex, race/ethnicity, pubertal status, indication for testing, and time of testing.

**Results:**

Subjects were 27 % female, with mean age of 8.9 years (SD 4.5); 75 % were pre-pubertal. Mean peak cortisol was 609.2 nmol/L (SD 213.0); mean delta cortisol was 404.2 nmol/L (SD 200.2). In separate multivariable models, weight, height, BSA and height z-score each remained independently negatively associated (*p* < 0.05) with peak and delta cortisol, controlling for indication of testing, baseline cortisol, and peak or delta ACTH, respectively. Age was negatively associated with peak but not delta cortisol in multivariable analysis.

**Conclusions:**

Despite the use of a weight-based dosing protocol, both peak and delta cortisol response to CRH are negatively associated with several measures of body size in children referred for clinical testing, raising the question of whether alternate CRH dosing strategies or age- or size-based thresholds for adequate cortisol response should be considered in pediatric patients, or, alternatively, whether this finding reflects practice patterns followed when referring children for clinical testing.

**Electronic supplementary material:**

The online version of this article (doi:10.1186/s13633-015-0018-y) contains supplementary material, which is available to authorized users.

## Background

Undiagnosed adrenal insufficiency can be life-threatening [1–3]. Children exposed to prolonged courses of exogenous glucocorticoids or with congenital or acquired forms of hypopituitarism are at increased risk for adrenocorticotropic hormone (ACTH) deficiency, or central adrenal insufficiency [1, 4]. Despite considerable clinical experience, the diagnosis of ACTH deficiency remains complex [5], and the “optimal” method to reliably diagnose ACTH deficiency remains unclear [6], particularly in children. While the insulin tolerance test (ITT) is often considered a “gold standard,” its use is limited due to the potential for severe hypoglycemia and its contraindication in patients with a history of seizures or cardiovascular disease [7, 8]. Although frequently used, the low-dose ACTH stimulation test does not allow for direct measurement of pituitary response, and concerns have been raised about the difficulty of reliably diluting the low dose of medication with precision [9]. The standard-dose ACTH stimulation test may be used to assess for primary adrenal insufficiency, but the large dose of 250 mcg produces supra-physiologic ACTH levels, which may lead to falsely reassuring cortisol responses in patients who may truly have inadequate responses to stress under more physiologic conditions [9].

Stimulation of the pituitary with corticotropin-releasing hormone (CRH), or corticorelin, can be used to test for both primary and secondary adrenal insufficiency through its stimulation of the release of ACTH from the pituitary [10–12]. The CRH stimulation test has been suggested as a useful and safe alternative to the ITT, as the cortisol response to CRH has been found to be significantly correlated with cortisol response to insulin-induced hypoglycemia [13, 14]. However, distinguishing between “healthy” and “inadequate” cortisol responses to this test remains a challenge, in part because there is not a clear consensus from previous studies on the patient-specific clinical factors that determine peak cortisol, particularly in children. Indeed, although some pediatric studies suggest that cortisol response after stimulation with CRH remains constant with increasing age [12, 15, 16], in other investigations, cortisol response after stimulation using other strategies, including low- [4] or standard-dose [17] ACTH, was negatively associated with age in children. The current recommended dosing for CRH is weight-based, which assumes a comparable pituitary and adrenal response to this medication across all ages and sizes. Previously published studies have not systematically focused on the relationship of body size to CRH response, particularly in children younger than six years [12, 15, 16]. Children under six years of age, in particular, may differ in clearance rates of medications due to incomplete maturation of physiologic and enzymatic processes [18]. Thus, the objective of the present study was to determine the effect of both age and body size on cortisol response, as measured by peak cortisol and cortisol rise from baseline (delta) to a standard CRH test in a cohort of nearly 300 children referred to a tertiary care center for suspicion of ACTH deficiency.

## Methods

### Design

This is a retrospective electronic medical record review of all children and adolescents referred for outpatient adrenal stimulation testing with CRH between January 2007 and April 2013 at The Children’s Hospital of Philadelphia Day Medicine Unit.

### Subjects

Subjects were less than 18 years of age at the time of testing; neonates (<30 days), most of whom receive clinically indicated adrenal stimulation testing as inpatients, were excluded. For subjects who underwent multiple stimulation tests, only the first was used for this analysis. All subjects underwent stimulation with 1 mcg/kg corticorelin (CRH) intravenously, prepared as a solution of 50 mcg corticorelin/mL by our institution’s main pharmacy. Per standard protocol at our institution, cortisol and ACTH were measured at baseline and 15, 30, 60, and 90 min after CRH administration. This study was reviewed, approved, and granted a waiver of consent by the Institutional Review Board of The Children’s Hospital of Philadelphia.

### Anthropometric and pubertal data

Height and weight were abstracted from electronic medical record as measured on the day of stimulation testing. If unavailable from the day of the test, heights were abstracted from the closest Endocrinology clinic visit that occurred no more than 3 months before or after stimulation. BSA was calculated using the Mosteller formula [19]. The following additional elements of the physical examination from the closest Endocrinology clinic visit within 3 months of stimulation testing were also abstracted: breast Tanner stage (girls only), testicular volume and Tanner stage (boys only), and Tanner stage for pubic hair (both girls and boys). Subjects without either height or weight data or without pubertal exam were excluded from further analysis; this was 54 out of 297 subjects initially identified to have completed testing.

### Laboratory assessment of ACTH and cortisol values

The main hospital laboratory at the Children’s Hospital of Philadelphia performed all laboratory testing. Cortisol and ACTH were measured by chemiluminescence. The lower limit of detection for cortisol was 1.0 mcg/dL (30 nmol/L) and for ACTH was 5 pg/mL (1 pmol/L). For the hospital’s main laboratory, the coefficient of variation for the cortisol assay was approximately 3–4 % and for ACTH was 5 %. (Personal communication with Tracey G. Polsky, MD, PhD, assistant director of the Clinical Chemistry Laboratory, The Children’s Hospital of Philadelphia, February 20, 2015) Subjects without available ACTH values were excluded from further analysis (*n* = 11).

### Indication for testing

**Fig. 1:**
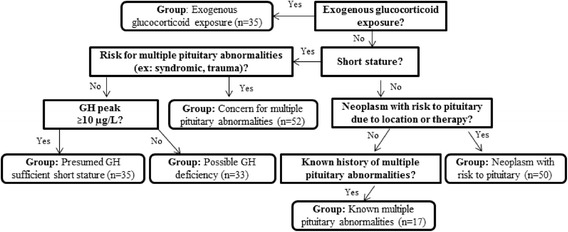
Categorization of indication for adrenal stimulation testing. Indications were extracted from outpatient Endocrinology notes; for many patients, more than one indication existed

All outpatient Endocrinology clinic visits within 3 months before or after stimulation testing were reviewed to determine the indication for referral for adrenal stimulation testing. A step-wise hierarchical approach was applied in order to assign a single, primary indication for each subject for the purpose of these analyses, even though patients could have more than one indication for testing. This is described here and illustrated graphically in Fig. 1. This categorization approach was developed based on a comprehensive review of pediatric adrenal insufficiency [1]. First, all subjects with exogenous glucocorticoid exposure noted as an indication for testing were assigned “exogenous glucocorticoid exposure” as their primary indication for testing. For remaining subjects, if short stature was listed as an indication for testing, they were categorized as either “concern for isolated growth hormone deficiency,” or “concern for multiple pituitary abnormalities (excluding neoplasm),” depending on whether the medical history or imaging suggested a possibility of multiple pituitary abnormalities. Many of these patients underwent CRH stimulation testing as well as growth hormone (GH) stimulation testing. Subjects who subsequently had a likely inadequate response to growth hormone stimulation testing (GH <10 mcg/L) [20] were classified into “possible growth hormone insufficiency.” Those with GH peak ≥ 10 mcg/L were considered to have “growth hormone sufficient short stature;” these subjects had apparently intact pituitary GH axis and no other indication of abnormal pituitary function aside from short stature. Next, the remaining subjects who did not have short stature listed as an indication for testing were categorized into one of the following groups: “neoplastic process with condition or therapy placing patient at risk for pituitary injury” or “known multiple pituitary abnormality.” No subjects were suspected of having primary adrenal insufficiency. Subjects without documentation of concern for central adrenal insufficiency as the indication for testing were excluded from further analysis (*n* = 10), for a final subject total of 222.

### Statistical analysis

“Peak cortisol” was defined as the maximum observed cortisol value measured following CRH administration. Change in cortisol, or “Δ cortisol,” was defined as the difference between the baseline and peak cortisol. Cortisol values below the detection limit of 1 mcg/dL were recorded as 1 mcg/dL for the purpose of data analysis. Units were converted to SI using the standard conversions of 27.59 nmol/L per 1.0 mcg/dL cortisol and 0.22 pg/mL per 1.0 pmol/L ACTH. Descriptive variables were summarized by mean ± standard deviation (SD), and outcome variables by mean ± standard error of the mean (SEM) unless otherwise stated. Categorical variables, including pubertal stage, were assessed across groups and sex using the chi-square test. Peak cortisol was assessed across weight and age quartiles using one-way ANOVA. Baseline, delta, and peak cortisol and ACTH were assessed across indication for testing using one-way ANOVA. Rate of peak cortisol < 500 nmol/L was compared across indications and age category (six years or younger vs older than six years) using the chi-square test. Two-sample t-test was used to compare the groups with suspected GH deficiency (those found to be likely GH sufficient and those likely to have GHD) and to compare cortisol response of subjects categorized by age younger than six years or older. Z-scores for height and BMI were calculated using CDC 2000 growth standards for children 2 years and older and WHO 2006 growth standards for children younger than 2 years, as recommended by the CDC [21]. To assess any potential confounding effect on the outcomes of interest (laboratory parameters of the CRH stimulation test, including peak cortisol, baseline cortisol, and Δ cortisol), bivariable linear regression analysis was first performed for covariates of interest, including: age, size (as measured by weight, height, BMI, BSA, BMI z-score, and height z-score), indication for testing, baseline cortisol, pubertal status, race/ethnicity, and time of day (AM or PM). Factors with p-value of <0.2 were included in multivariable linear regression models for peak and delta cortisol. Next, backward elimination using *p* < 0.1 was used to determine final multivariable models for peak and delta cortisol. Each model included only one “size factor” (i.e., weight, height, BMI, or BSA) or age due to the strong positive correlation (*p* < 0.0005) between age and each of weight, height, BMI, and BSA. BMI z-score and height z-score were also included in separate models because z-scores were determined using both age and absolute height or BMI. Thus, to avoid the collinearity problem, only one of each of age or “size factors” was included per model. Of note, weight, height, BMI, BSA are absolute size factors, while BMI z-score and height z-score are calculated based on reference values for age and sex, and thus are relative size factors.

Data analysis was performed with Stata, Release 13.0 (College Station, TX) and R version 3.0.0. A two-sided *p* value of <0.05 was considered statistically significant.

## Results

### Subjects

Table 1 shows subject characteristics, summarized by indication for testing. The 222 subjects (27 % female) who met inclusion criteria had a mean age of 8.9 years (SD 4.5, range 0.4–17.8 years). Seventy-five percent were pre-pubertal (Tanner I), 22 % were peri-pubertal (Tanner II-IV), and 4 % were post-pubertal (Tanner V). These proportions were not significantly different between boys and girls (*p* = 0.07). Seventy-nine percent of subjects did not have pubic hair at time of stimulation testing. By body mass index (BMI) z-score, 26 % of subjects were overweight or obese (BMI z-score ≥1.04) [21]. As expected in a population including glucocorticoid-treated children, as well as those with known pituitary abnormalities and/or clinically referred for short stature, subjects were relatively short (mean height z-score −1.96, range −6.07 to 3.2). Subjects with known multiple pituitary abnormalities tended to be older and weighed more than those who were undergoing initial evaluation for pituitary abnormalities (*p* < 0.0005 for both; see Table 1). Additionally, for each group, the majority of subjects were male. This was most notable for the group with neoplasms with risk to the pituitary. The two groups of subjects screened for growth hormone deficiency (GHD) were similar in age, weight, height, and gender distribution (*p* > 0.05), and 49 % of those tested for GHD had peak growth hormone of < 10 mcg/L.

**Table 1:** Subject characteristics by indication for testing

Indication for testing	Presumed GH sufficient short stature (GH ≥ 10 μg/L)	Possible GHD (GH < 10 μg/L)	Concern for multiple pituitary abnormalities	Neoplasm with risk to pituitary	Exogenous glucocorticoid exposure	Known multiple pituitary abnormalities	*p*-value^a^
n	35	33	52	50	35	17	-
Sex (% female)	34 %	27 %	29 %	16 %	34 %	29 %	0.4
Age (years)	9.1 (4.6)	8.4 (4.1)	7.3 (5.0)	10.1 (3.0)	8.1 (4.9)	12.6 4.2)	0.0002
Tanner stage (Breast/Genital)							
I	28 (80 %)	29 (88 %)	44 (85 %)	33 (66 %)	25 (71 %)	7 (41 %)	<0.0005
II–IV	7 (20 %)	4 (12 %)	7 (13 %)	17 (34 %)	6 (17 %)	7 (41 %)	
V	0	0	1 (2 %)	0	4 (11 %)	3 (18 %)	
Presence of pubic hair							
No	29 (83 %)	32 (97 %)	47 (90 %)	32 (64 %)	27 (77 %)	8 (47 %)	<0.0005
Yes	6 (17 %)	1 (3 %)	5 (10 %)	18 (36 %)	8 (23 %)	9 (53 %)	
Weight (kg)	23.6 (11.1)	24.5 (14.2)	25.9 (17.4)	31.8 (13.1)	31.0 (22.9)	47.5 (22.4)	<0.0005
BMI z-score	−0.50 (0.98)	−0.04 (1.25)	0.55 (1.12)	0.27 (1.05)	0.45 (1.36)	0.57 (1.53)	0.0009
Height z-score	−2.8 (0.8)	−2.6 (0.9)	−1.8 (1.6)	−1.6 (1.4)	−1.4 (1.3)	−1.6 (1.5)	<0.0005

Data are presented as mean (SD)

^a^As determined by ANOVA across indication for testing

### Cortisol and ACTH response to stimulation

Mean peak cortisol for all subjects was 609.2 nmol/L (SD 213.0, range 27.59–1404.3). Mean Δ cortisol was 404.2 nmol/L (SD 200.2, range 0–905.0). Using cortisol of 500 nmol/L, a commonly used threshold to define “failure” to achieve reassuring cortisol response to CRH stimulation [6, 22], failure rate varied significantly by indication for testing (*p* = 0.0066 by ANOVA). Fourty-eight (22 %) of all subjects had peak cortisol less than 500 nmol/L. The greatest failure rate occurred in the group tested due to exogenous glucocorticoid exposure; this group had 63 % (22/35) of subjects with peak cortisol < 500 nmol/L.

Mean peak ACTH for all subjects was 20.2 pmol/L (SD 18.7, range 1.1–197.8). Mean baseline ACTH was 4.1 pmol/L (SD 3.6, range 1.1–30.4); mean delta ACTH was 16.1 pmol/L (SD 18.2, range 0–192.5). Mean peak ACTH for subjects with peak cortisol less than 500 nmol/L was 11.4 pmol/L (SD 9.0, range 1.1–36.3), compared to mean peak ACTH of 22.6 pmol/L (SD 19.9, range 3.6–197.8) for subjects with peak cortisol of 500 nmol/L or greater. This difference was statistically significant (*p* = 0.0002 by two-sample t-test).

### Relationship between cortisol response, body size, age, and other clinical covariates

Table 2 displays results of bivariable analysis of factors predicted to have a potential effect on peak or delta cortisol. In bivariable analysis, peak cortisol was significantly negatively associated with age, weight, height, BMI, BSA, and height z-score (*p* < 0.05 for each). Negative associations between body size factors and delta cortisol were also found but were less robust, with only height z-score reaching a similar level of statistical significance (*p* < 0.05). For purposes of further investigation of the relationship between outcomes with the predictive factors, factors with p-value < 0.2 were included in multivariable analysis and noted in Table 2, as described in Methods; for delta cortisol, these factors included weight, height, and BSA. Unlike for peak cortisol, age was not correlated with delta cortisol (*p* > 0.2). Baseline cortisol was significantly positively correlated with peak cortisol and negatively correlated with delta cortisol (*p* < 0.0005 for each). Baseline ACTH (*p* = 0.007), delta ACTH (*p* < 0.0005), and peak ACTH (*p* < 0.0005) were significantly positively correlated with peak cortisol. Delta and peak ACTH were also significantly positively correlated with delta cortisol (*p* < 0.0005 for each) (Table 2). To assess for bias conferred by subjects considered to have central cortisol deficiency, sensitivity analysis was performed. In bivariable analysis excluding subjects with peak cortisol < 500 nmol/L, age and size variables of interest (weight, height, BSA, and height z-score) remained significantly negatively associated with peak cortisol (*p* < 0.05). BMI also remained negatively associated with peak cortisol (*p* = 0.13) (data not shown).

**Table 2:** Bivariable analysis of peak and delta cortisol

	Peak cortisol (nmol/L)		Delta cortisol (nmol/L)	
	Coefficient	*p*-value	Coefficient	*p*-value
Age (years)	−7.6	0.02*		NS
Weight (kg)	−2.5	0.002 **	−1.4	0.06#
Weight inverse (1/kg)	1153.9	0.004 **		NS
Height (m)	−174.1	0.001 **	−85.9	0.08#
BMI (kg/m^2^)	−6.5	0.04*		NS
BSA (m^2^)	−123.8	0.001 **	−67.4	0.05#
BMI z-score	−14.6	NS		NS
Height z-score	−41.7	<0.0005**	−33.4	<0.005**
Sex (vs female)	−42.8	0.18#	−19.1	NS
Race		NS		NS
Ethnicity (Hispanic/Latino vs Non-Hispanic/Latino)		NS	−94.3	0.09#
Pubertal status (vs Tanner I)		0.21		0.09#
Tanner II-IV		NS		NS
Tanner V	−115.2	0.14#	−154.7	0.03*
Pubic hair development (yes vs no)		NS		NS
Indication for testing (vs GH sufficient short stature)		<0.0005**		<0.0005**
GHD		NS		NS
Concern for multi-pituitary abnormalities	−65	0.11#	−55.1	0.16
Neoplasm with risk to pituitary		NS		NS
Exogenous glucocorticoid exposure	−312.7	<0.0005**	−239.0	<0.0005**
Known multi-pituitary abnormalities	−133.7	0.02*	−105.3	0.05
Time of testing (PM vs AM)	−40.1	0.17#	−62.2	0.02*
Baseline cortisol (nmol/L)	0.72	<0.0005**	−0.35	<0.0005**
Baseline ACTH (pmol/L)	10.6	0.01*	−5.9	0.11#
Delta ACTH (pmol/L)	3.4	<0.0005**	4.0	<0.0005**
Peak ACTH (pmol/L)	3.6	<0.0005**	3.6	<0.0005**

#*p*-value <0.2 (for use in multivariate analysis); *p-value;<0.05, **p-value < 0.005

Other factors with marginal significance (*p* < 0.2) in bivariable analysis of peak cortisol were sex (male vs female, *p* = 0.182) and time of testing (after vs before 12:00 PM, *p* = 0.17); both factors were negatively associated with peak cortisol. For delta cortisol, ethnicity (Hispanic/Latino vs non-Hispanic/Latino, *p* = 0.09) and time of testing (after vs before 12:00 PM, *p* = 0.023) were significantly negatively correlated. Pubertal status (post-pubertal vs pre-pubertal: *p* = 0.003) was significantly negatively correlated with delta cortisol but not peak cortisol. These factors were included in initial multivariable models for peak and delta cortisol.

For peak and delta cortisol, indication for testing was associated with cortisol response (*p* < 0.0005). To account for this in multivariable linear regression, interaction terms between indication for testing and the size variable of interest were created and included in each model.

### Multivariable model for peak cortisol response to stimulation

Multivariable linear regression analysis was used to determine factors that were independently associated with cortisol response to CRH stimulation. Table 3 displays final multivariable linear regression models obtained after backward elimination of non-significant (*p* > 0.1) variables that were initially included from bivariable analysis. Baseline cortisol and peak ACTH remained significantly positively associated with peak cortisol. Sex was marginally associated with peak cortisol in each multivariable model, with p-values ranging from 0.048 for the model including age to 0.057 for the model including height z-score. Each model also included interaction terms between indication for testing and either age or the size factor of interest. Because age and size are highly associated, only one of these was included in each model to avoid collinearity, as described in Methods.

**Table 3:** Multivariable models for peak cortisol

	Age (years)		Weight (kg)		Height (m)		BSA (m^2^)		Height z-score	
	Coefficient	*p*-value	Coefficient	*p*-value	Coefficient	*p*-value	Coefficient	*p*-value	Coefficient	*p*-value
Age or size factor	−6.6	0.01*	−5.9	0.01*	−135.3	0.001 **	−221.3	0.01*	−70.8	0.02*
Sex (vs female)	−46.4	0.05*	−45.7	0.05	−45.1	0.05	−45.2	0.06		
Indication for testing (vs GH sufficient short stature)										
GHD		NS		NS		NS		NS		NS
Concern for multi-pituitary abnormalities	−67	0.05*	−137	0.06	−63	0.06	−173	0.07		NS
Neoplasm with risk to pituitary		NS		NS		NS		NS		NS
Exogenous glucocorticoid exposure	−249	<0.0005**	−398	<0.0005**	−242	<0.0005**	−425	<0.0005**		NS
Known multi-pituitary abnormalities	−104	0.02*	−244.0	0.03*	−94	0.04*		NS		NS
Interaction variables (vs GH sufficient group*age or size factor)										
GHD*(age or size factor)				NS				NS		NS
Concern for multi-pituitary*(age or size factor)				NS				NS		NS
Neoplasm*(age or size factor)				NS				NS		NS
Exogenous glucocorticoids*(age or size factor)			6.4	0.02*			212	0.05*	129.3	<0.0005**
Known multi-pituitary*(age or size factor)				NS				NS		NS
Baseline cortisol (nmol/L)	0.59	<0.0005**	0.60	<0.0005**	0.58	<0.0005**	0.60	<0.0005**	0.65	<0.0005**
Peak ACTH (pmol/L)	2.56	<0.0005**	2.49	<0.0005**	2.47	<0.0005**	2.49	<0.0005**	2.20	<0.0005**
R^2^, n	0.5092, 222		0.5201, 222		0.5182, 222		0.5196, 222		0.5481, 222	

*p-value <0.05, **p-value < 0.005 by multivariate linear regression

Models for weight, height z-score, and BSA included significant interaction terms between indication for testing and size. For these models, an interaction between size and exogenous glucocorticoid administration was detected (*p* < 0.05); within glucocorticoid-exposed children, the smallest children seemed to have the lowest peak cortisol, as described in more detail below. Multivariable linear regression analysis was repeated separately for the group with exogenous glucocorticoid exposure (Additional file 1: Table S1). In this analysis, the exogenous glucocorticoid group did not have an independent association between peak cortisol and weight or BSA (*p* > 0.05), but did have a significant positive association between peak cortisol and height z-score (beta = 58.6, *p* = 0.015), opposite the direction of the negative association between size and peak cortisol over all other subjects.

### Multivariable model for delta cortisol

Table 4 displays final multivariable linear regression models obtained after backward elimination of non-significant (*p* > 0.1) variables that were initially included from bivariable analysis with delta cortisol. In the final multivariable models, pubertal status, ethnicity, and time of stimulation testing were no longer significantly independently associated with delta cortisol. Baseline cortisol remained significantly negatively and delta ACTH significantly positively associated with delta cortisol.

**Table 4:** Multivariable models for delta cortisol

	Weight		Height		BSA (m^2^)		Height z-score	
	Coefficient	*p*-value	Coefficient	*p*-value	Coefficient	*p*-value	Coefficient	*p*-value
Size factor	−5.8	0.02*	−128.3	0.003 **	−216.8	0.02	−71.5	0.02*
Indication for testing (vs GH sufficient short stature)								
GHD		NS		NS		NS		NS
Concern for multi-pituitary abnormalities		NS		NS		NS		NS
Neoplasm with risk to pituitary		NS		NS		NS		NS
Exogenous glucocorticoid exposure	−429	<0.0005**	−256	<0.0005**	−472	<0.0005**		NS
Known multi-pituitary abnormalities	−230.0	0.05*	−96	0.05*		NS		NS
Interaction variables (vs GH sufficient group*size factor)								
GHD*(size factor)		NS				NS		NS
Concern for multi-pituitary*(size factor)		NS				NS		NS
Neoplasm*(size factor)		NS				NS		NS
Exogenous glucocorticoids*(size factor)	7.0	0.01*			245.5	0.03*	159	<0.0005**
Known multi-pituitary*(size factor)		NS				NS		NS
Baseline cortisol (nmol/L)	−0.44	<0.0005**	−0.46	<0.0005**	−0.44	<0.0005**	−0.40	<0.0005**
Delta ACTH (pmol/L)	2.47	<0.0005	2.49	<0.0005**	2.49	<0.0005**	2.22	<0.0005**
R^2^, n	0.3956, 222		0.3875, 22		0.3947, 222		0.4621, 222	

*p-value <0.05, **p-value < 0.005 by multivariate linear regression

Similar to the models for peak cortisol, each model of delta cortisol included interaction terms between indication for testing and the size factor of interest, as described above. Models for weight, BSA, and height z-score included significant interaction terms between indication for testing and size. Again, for these models, the association between delta cortisol and size (weight, BSA, or height z-score) among subjects tested due to exogenous glucocorticoids was positive, opposite that of overall subjects. Similar to the findings for peak cortisol, when analysis of delta cortisol was repeated by indication for testing, a significant positive association (*p* = 0.003) between height z-score (but not weight or BSA) and delta cortisol was found for subjects with exogenous glucocorticoid exposure (data shown in Additional 2: Table S2).

### Relationship between weight, age and peak cortisol response

**Fig. 2:**
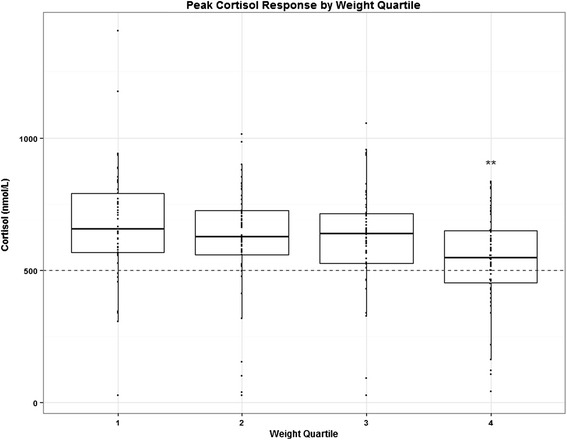
Peak cortisol response to CRH stimulation by weight quartile over the entire cohort. *N* = 222 (quartile 1: *n* = 57, quartile 2: *n* = 54, quartile 3: *n* = 56, quartile 4: *n* = 55). Dotted line represents a commonly used threshold for stimulation test failure, cortisol of 500 nmol/L. ** p-value = 0.003 for quartile 4 vs quartile 1 after Bonferroni correction

Figure 2 displays peak cortisol response by quartiles of absolute weight. As shown, subjects in the highest weight quartile tended to have the lowest peak cortisol, consistent with the negative correlation found on multivariable regression. By one-way ANOVA, peak cortisol differed significantly across weight quartiles (*p* = 0.0076). In the highest weight quartile, 36 % (20/55) of subjects failed to achieve a peak cortisol of 500 nmol/L, as opposed to 17 % (28/167) of subjects in quartiles 1–3 (*p* = 0.002 by chi-square test). To better understand the interaction between weight, age, and cortisol response, this analysis was repeated by age quartile, and no significant difference in peak cortisol across age quartiles was noted (*p* > 0.05, data not shown).

### Relationship between peak and baseline cortisol and time of testing

Although mean baseline cortisol drawn between 8:00 and 9:00 AM tended to be higher than those drawn after 9:00 AM (226.2 nmol/L, SD 88.0 for 12 subjects vs 200.2 nmol/L, SD 148.2 for 206 subjects), this did not reach statistical significance (*p* = 0.5). Peak cortisol also did not differ significantly between these groups (mean peak 562.8 nmol/L, SD 171.4 vs 610.0 nmol/L, SD 216.6, *p* = 0.5). Similarly, when divided into subjects tested before and after noon, baseline cortisol did not differ significantly (192.0 nmol/L, SD 106.6 for 117 subjects vs 212.9 nmol/L, SD 180.3 for 101 subjects, *p* = 0.3).

### Baseline and peak cortisol response in children six years or younger

We sought to characterize cortisol response in children 6 years and younger, as limited data is available for children of this age referred for clinical testing. Baseline cortisol was significantly higher in children 6 years or younger (239.4 nmol/L, SD 172.0 for 57 subjects vs 188.7 nmol/L, SD 132.5 for 165 subjects, *p* = 0.0223 by two-sample t-test). Peak cortisol, however, did not significantly differ between these groups (652.1 nmol/L, SD 221.8 for 57 subjects vs 594.4 nmol/L, SD 208.5 for 165 subjects, *p* = 0.08). Rate of peak cortisol response less than 500 nmol/L also did not differ significantly between these groups (11/57 (19 %) vs 37/165 (22 %), *p* = 0.6 by chi-square).

### Relationship between weight, indication for testing, and peak cortisol

**Fig. 3:**
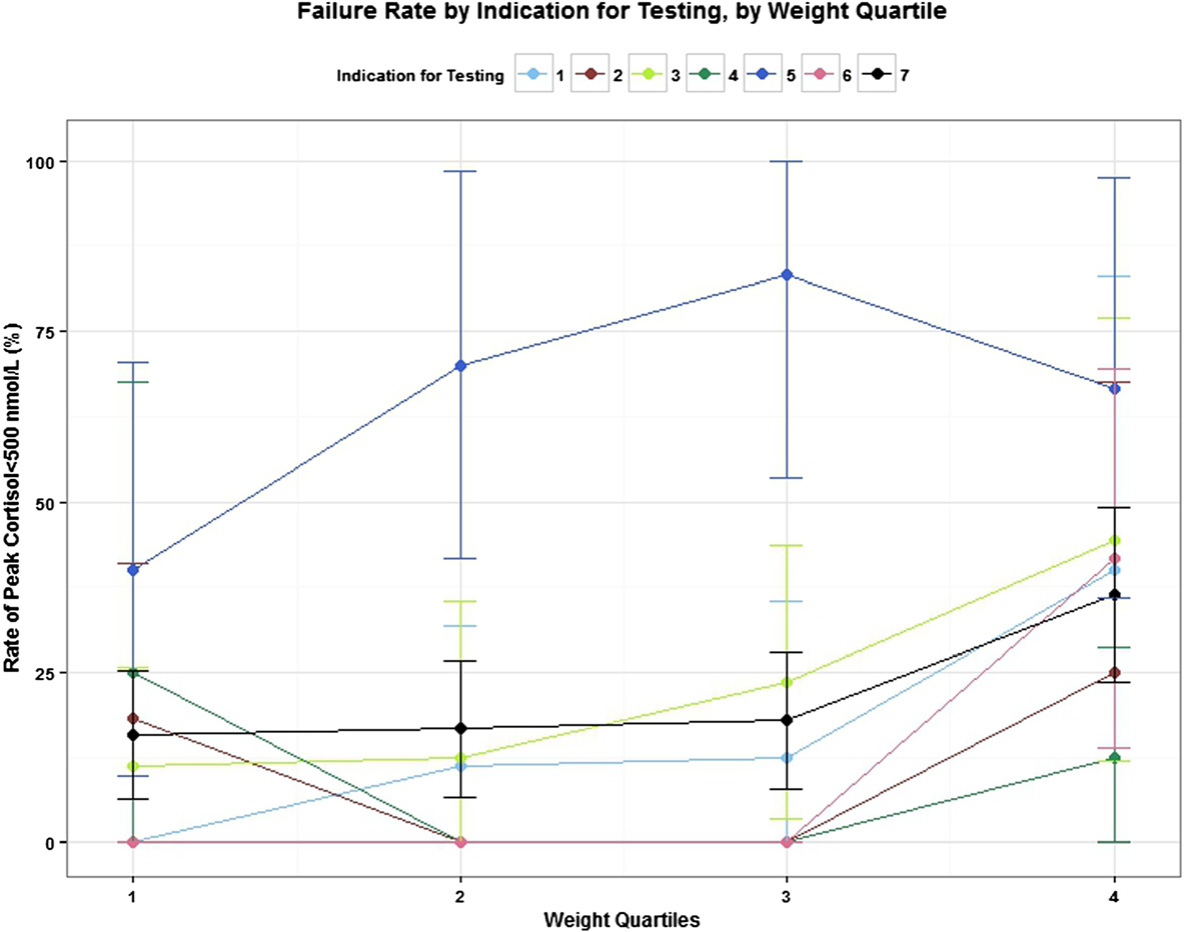
Failure rate (rate of peak cortisol < 500 nmol/L), by absolute weight quartile and indication for testing. Error bars represent 95 % confidence intervals. Total subjects are represented by the black line. Indication for testing was numbered as follows: 1. GH sufficient short stature (*n* = 35); 2. Probable GHD (*n* = 33); 3. Concern for multiple pituitary abnormalities (*n* = 52); 4. Neoplasm with risk to pituitary (*n* = 50); 5. Exogenous glucocorticoid exposure (*n* = 35); 6. Known multiple pituitary abnormalities (*n* = 17); 7. All indications (*n* = 222)

To better understand the interaction between weight and indication for testing in the multivariable model for peak cortisol, subjects who “failed” (peak cortisol < 500 nmol/L) CRH stimulation were compared across weight quartiles and indication for testing, as shown in Fig. 3. As shown, the group with exogenous glucocorticoid exposure had significantly higher rates of failure, particularly for the middle two weight quartiles. A summary of failure rates for all groups is shown in black; this demonstrates the trend across groups toward higher failure rates among the highest weight quartile. Overall failure rates for each indication for testing are summarized in Table 5.

**Table 5:** Cortisol and ACTH response by indication for testing

Indication for testing	Presumed GH sufficient short stature (GH ≥ 10 μg/L)	Possible GHD (GH < 10 μg/L)	Concern for multiple pituitary abnormalities	Neoplasm with risk to pituitary	Exogenous glucocorticoid exposure	Known multiple pituitary abnormalities	*p*-value^a^
n	35	33	52	50	35	17	
Baseline cortisol (nmol/L)	231.0 (124.3)	203.3 (136.6)	221.1 (182.0)	199.1 (119.4)	145.7 (139.8)	202.2 (139.6)	0.18
Delta cortisol (nmol/L)	454.9 (168.1)	485.2 (139.7)	399.8 (160.4)	470.2 (189.2)	215.9 (218.4)	349.6 (219.5)	<0.0005
Peak cortisol (nmol/L)	685.9 (161.8)	688 .5 (179.8)	620.9 (178.6)	675.8 (138.5)	373.2 (246.1)	552.2 (227.0)	<0.0005
Baseline ACTH (pmol/L)	3.2 (1.8)	5.9 (6.6)	4.2 (2.6)	3.6 (1.9)	3.7 (4.1)	4.7 (2.8)	0.036
Delta ACTH (pmol/L)	14.9 (9.3)	23.0 (18.5)	13.2 (11.6)	21.4 (26.6)	6.3 (4.9)	18.3 (25.6)	0.0007
Peak ACTH (pmol/L)	18.1 (9.6)	28.9 (17.9)	17.4 (11.5)	25.0 (27.0)	10.1 (7.5)	23.0 (26.3)	0.0003
Failure Rate (peak cortisol < 500 nmol/L)	11.4 %	9.1 %	21.2 %	6.0 %	62.9 %	29.4 %	<0.0005

Data are presented as mean (SD)

^a^As determined by ANOVA across indication for testing for cortisol and ACTH values; by Chi-square for Failure Rate

Finally, to minimize effects of indication of testing on cortisol response, bivariable analysis was repeated for the two groups with the most similar subjects: those tested due to concern for GHD and subsequently found to be either likely GH sufficient or deficient. These groups had similar weight and age distributions (*p* = 0.79 and *p* = 0.50 by two-sample t test). In this analysis, the negative association between weight and peak cortisol (*p* = 0.008) and age and peak cortisol (*p* = 0.021) persisted, suggesting that the differences among subjects due to indication for testing cannot solely explain the negative correlation between body size or age and cortisol response.

## Discussion

In both bivariable and multivariable analyses, peak cortisol after CRH stimulation testing was significantly negatively associated with age and multiple measures of body size, including weight, height, height z-score, and BSA in our study of over 200 children referred for clinical testing. Delta cortisol, another measure of cortisol response to stimulation, was similarly negatively associated with weight, height, height z-score, and BSA, but was not significantly associated with age.

These findings may be interpreted in several ways. First, due to the retrospective nature of our study, referral bias may have played a role. For example, the high failure rate among glucocorticoid-exposed subjects may be due to referral of the most severely affected individuals, raising the pre-test probability of failure. Additionally, younger (and smaller) subjects may have been referred more readily despite their relatively healthy clinical status, making these subjects more likely to pass their stimulation test. Another possibility is the changing nature of indication for testing across age and size. For example, older and larger subjects may have been tested for indications that also increased their pre-test probability of failure, independent of their size and age. To account for these possibilities, we performed several sensitivity analyses. As explained above in Results, when bivariable analysis was repeated only for subjects tested due to concern for isolated growth hormone deficiency, the negative association between peak cortisol and weight or age persisted. Therefore, another important possible explanation to explore is that true physiologic differences in adrenal response to CRH exist, despite weight-based dosing, across the wide range of body sizes and ages of children included in the present study.

An important previous study in healthy children did not find age- or size-based differences in pharmacokinetic or pharmacodynamic parameters in the response to CRH, but the sample size was relatively small (*n* = 21, girls and boys ages 6–15 years), and these investigators themselves noted the lack of data for children under 6 years of age [16]. However, in investigations using other techniques to assess hypothalamic-pituitary-adrenal axis function (low- and standard-dose ACTH), peak cortisol response in healthy children [17] and in children exposed to exogenous glucocorticoids [4] did decrease with age, consistent with our findings using CRH stimulation testing over all subjects. As noted previously, the subjects in our study with glucocorticoid exposure did not demonstrate a similar negative relationship between peak or delta cortisol and height z-score (Additional file 1: Table S1 and Additional file 2: Table S2). This may reflect the higher likelihood of adrenal suppression in subjects who had glucocorticoid exposure great enough to negatively affect height growth, as even inhaled corticosteroids have been associated with decreased height growth, particularly in prepubertal children [23].

Other studies have also found associations between cortisol levels and age in healthy children, but the direction of effect has differed between studies depending on statistical approaches, highlighting how the simultaneous effects of age and size are challenging to disentangle [24–27]. For example, in one of these investigations, salivary cortisol concentration was initially found to increase with age, but after statistical adjustment for BSA, the relationship with age seemed reversed [25]. The authors posit that this finding could reflect a lower production rate or higher rate of clearance of cortisol with age [25]. A separate study focused on this question found that daily cortisol production remained constant with age, again after adjustment for body surface area [28]. Taken together, these studies illustrate the important challenge in pediatrics of scaling for size when interpreting experimental results across a wide range of subject ages [29], particularly in the youngest children, in whom there is the additional complexity of incomplete maturation of kidney and liver function, which also affects drug metabolism [30].

To our knowledge, an independent, negative relationship between cortisol response to CRH and body size has not been demonstrated previously. As discussed above, due to the high correlation between age and body size, discerning the relative effects on cortisol response of each of these is a challenging undertaking, particularly due to differences in indication for testing across age and weight. For example, the association between peak cortisol response and age/size may be due to maturational differences in the responsiveness of the adrenal gland to ACTH and/or clearance of cortisol, differences that cannot be fully adjusted by the current weight-based dosing regimen of CRH.

Differences in adrenal gland size may also partly explain differences in responsiveness across the ages and body sizes tested. The adrenal gland does not grow at the same pace as the rest of the body; instead, it decreases in size from birth to around one year of age, then gains mass, but more slowly than the body as a whole [31]. If circulating cortisol concentration were to remain constant or even increase with age as has been described by several authors [24–26], these relatively smaller adrenal glands would need to produce proportionally more cortisol to distribute across relatively larger blood volumes, assuming constant clearance. Although these relatively smaller adrenal glands would thus produce larger amounts of cortisol relative to body size on a constant basis, they may not produce as robust a response to acute stimulation, as they may already be operating at a “higher capacity.” This “lower reserve” could explain the lower peak cortisol response to stimulation in subjects with larger body surface area and relatively smaller adrenal glands.

Alternatively, age may be the driving force in the negative association, through mechanisms not primarily driven by body size. We looked for but did not find an effect of puberty and/or presumed adrenarche (pubic hair development alone) on cortisol response, but the sample was enriched in young, pre-pubertal children, so these effects may have been more difficult to detect. Indeed, at least one previous study has suggested increased volume of distribution and more rapid clearance of cortisol with the onset of puberty [32]. Sampling beyond the usual prescribed time range for CRH stimulation testing would be required to estimate these parameters. Additional careful pharmacokinetic and pharmacodynamics studies in children could help answer these questions.

### Strengths and limitations

The strengths of the present study include its large size and wide range of ages studied, including 57 children age six years and younger, the largest study to our knowledge of children in this age range who have undergone stimulation with CRH. As mentioned above, our study has limitations related to its retrospective nature. One potential limitation was the health status of our subjects, who had a wide range of diagnoses and exposures to medications and therapies. Although this limits our ability to generalize to healthy children, our subjects are representative of the patients who most often undergo adrenal stimulation testing. As noted above, however, 89 % of subjects with presumed growth hormone sufficient short stature reached a cortisol peak of 500 nmol/L, consistent with our belief that this group was representative of subjects with a likely intact HPA axis, regardless of their short stature. In addition, we considered the possibility that age- or size-related differences in indication for testing could introduce bias into our results. We observed the negative association between peak cortisol and age or size even in multivariable regression analyses including testing indication and interaction terms between testing indication and age/size (Table 3). However, it would be optimal to reproduce these results in additional cohorts prospectively grouped by age and indication for testing and to consider studies in healthy children as well. Additionally, referral patterns may be valuable to study, as one interpretation of our results may be that younger/smaller children with intact adrenal function may be more likely to undergo testing to exclude ACTH deficiency as part of an initial evaluation. This may explain our finding that smaller, shorter children tended to have higher peak cortisol, opposite of what one would expect if these children were short due to underlying pathology associated with ACTH deficiency. Finally, an additional limitation is that cumulative glucocorticoid exposure was unavailable for analysis; although this was not the primary focus of our study, it may have allowed for a better understanding of the cortisol response among this group of subjects.

The present study, the largest collection to date of pediatric CRH stimulation testing results to our knowledge, demonstrates that cortisol response to CRH stimulation is negatively associated with both age and size, as reflected by weight, height, BSA, and height z-score, in children referred for clinical testing, even after accounting for important clinical covariates. Additional careful pharmacokinetic and pharmacodynamic studies, including serial measurements of CRH, ACTH, and cortisol, could help clarify the etiology of these differences. That is, the volume of distribution of CRH, and/or the clearance of cortisol are at least two potential sources of age- or size-related variation.

## Conclusions

Our results suggest that to better interpret peak cortisol response across a wide range of ages and sizes in the pediatric population, it may be helpful to consider the possibility of greater cortisol responses in the youngest and smallest patients. Specifically, for patients with “borderline” peak cortisol response, it may be helpful to consider the patient-specific characteristics of age and size when determining whether the patient has “passed” or “failed” the stimulation test. Our study was limited to a population referred for clinical testing, but the potential for generalizability of these findings make future prospective studies focused on size and age very important. Optimally, prospective development of age- or size-dependent thresholds for cortisol response might increase the clinical utility of this provocative test, particularly in the youngest at-risk patients.

## Additional files

Additional file 1: Table S1. Sub-analysis of interaction terms in multivariable models for peak cortisol. Description: Separate analysis of groups with significant interaction terms in multivariable models. This displays multivariable models of weight, BSA, and height for the group exposed to exogenous glucocorticoids only. (DOC 32 kb) Additional file 2: Table S2. Sub-analysis of interaction terms in multivariable models for delta cortisol. Description: Separate analysis of groups with significant interaction terms in multivariable models. This displays multivariable models of weight, BSA, and height for the group exposed to exogenous glucocorticoids only. (DOC 31 kb)

## Abbreviations

CRH: Corticotropin-releasing hormone, corticorelin
ACTH: Adrenocorticotropic hormone
BSA: Body surface area
ITT: Insulin tolerance test
GH: Growth hormone
GHD: Growth hormone deficiency
BMI: Body mass index
HPA: Hypothalamic-pituitary-adrenal
