# Supplementary material for: Peak cortisol response to corticotropin-releasing hormone is associated with age and body size in children referred for clinical testing: a retrospective review
Source: Int J Pediatr Endocrinol. 2015 Oct 22;2015:22. doi: 10.1186/s13633-015-0018-y (PMC4618529; doi:10.1186/s13633-015-0018-y)
Supplement: Additional file 2: Table S2. — Sub-analysis of interaction terms in multivariable models for delta cortisol. Description: Separate analysis of groups with significant interaction terms in multivariable models. This displays multivariable models of weight, BSA, and height for the group exposed to exogenous glucocorticoids only. (DOC 31 kb) [file 13633_2015_18_MOESM2_ESM.doc]

Additional file 2

| **Additional Table 2. Sub-analysis of interaction terms in multivariable models for delta cortisol, exogenous glucocorticoid exposure group only** | | | | | | | | | |
| --- | --- | --- | --- | --- | --- | --- | --- | --- | --- |
|  | **Weight (kg)** | |  | **BSA (m^2^)** | |  | **Height z-score** | | |
|  | **Coefficient** | **p-value** |  | **Coefficient** | **p-value** |  | **Coefficient** | **p-value** |  |
| **Size Factor** | -0.19 | 0.893 |  | -20.0 | 0.77 |  | 71.8 | 0.003 | ** |
| **Delta ACTH (pmol/L)** | 31 | <0.0005 | ** | 31.2 | <0.0005 | ** | 24.9 | <0.0005 | ** |
| **Baseline cortisol (nmol/L)** | -0.6 | 0.019 | * | -0.61 | 0.018 | * | -0.38 | 0.088 |  |
| **R^2^, n** | 0.3983, 35 | |  | 0.3996, 35 | |  | 0.5490, 35 | |  |
| *p-value <0.05, **p-value<0.005 by multivariate linear regression | | | |  |  |  |  |  |  |
